# δ‐Bismuth Oxide Responsible for Tooth Discolouration—A Laboratory Investigation

**DOI:** 10.1111/iej.70077

**Published:** 2025-12-01

**Authors:** J. Camilleri, P. Zaslansky, N. Ramanan

**Affiliations:** ^1^ Dentistry, School of Health Sciences, College of Medicine and Health University of Birmingham Birmingham UK; ^2^ Department for Operative and Preventive and Paediatric Dentistry, Centrum für Zahn‐, Mund‐Und Kieferheilkunde Charité—Universitätsmedizin Berlin Berlin Germany; ^3^ Diamond Light Source Harwell Science and Innovation Campus Didcot UK

**Keywords:** bismuth oxide, chemical characterisation, phase changes, sodium hypochlorite, synchrotron, tooth discolouration

## Abstract

**Aims:**

To investigate the phase changes of bismuth oxide in contact with sodium hypochlorite responsible for tooth discolouration.

**Methodology:**

Bismuth oxide (monoclinic α−phase; C) was mixed with sodium hypochlorite at 20°C, 37°C, and 60°C (B20, B37, B60) for a period of 24 weeks with weekly refreshing of solutions. The products were imaged by scanning electron and optical microscopy and characterized by thermographic analysis (TGA), phase analysis by X‐ray diffraction (XRD) using Bragg Brentano geometry and Pilatus detector, infrared spectroscopy (FT‐IR), and X‐ray absorption fine structure (XAFS).

**Results:**

The interaction of bismuth oxide with sodium hypochlorite resulted in a change in microstructure and colour. The thermographic assessment showed a change in mass (5%–10% weight change) and colour reversal to the yellow bismuth oxide at ~450°C. Phase changes dependent on temperature were demonstrated with δ‐bismuth oxide, sodium bismuthate and bismuth oxychloride formed as by‐products at the different temperatures.

**Conclusions:**

The δ‐phase bismuth oxide formation led to the material darkening which will cause tooth discolouration in a clinical setting. Due to the phase changes, the material chemistry after the interaction is different from that of the material placed in the tooth. The by‐products of the reaction have not been tested for use in patients. It is recommended to ban the use of bismuth oxide from dental materials and other clinical use due to its instability. The clinical guidance for endodontic treatment needs to be changed to reflect this.

## 1 Introduction

There is no medico‐legal requirement for enhanced radiopacity for dental materials and devices. The Medical Devices Regulation (EU) 2017/745 and (EU) 2017/746 (European Commission 2023) and clinical guidelines (Council on Dental Materials, Instruments, and Equipment 1981) mandate radiopacity for diagnostic purposes. Sufficient radiopacity is crucial to distinguish restorative materials from surrounding tooth structure or pathosis on radiographs, which is essential for proper patient care, informed consent, and avoiding misdiagnosis, thus contributing to medico‐legal safety. Bismuth‐based materials are used frequently in medicine and dentistry to enhance the radiopacity of dressings such as bismuth iodoform paraffin paste gauzes and materials such as mineral trioxide aggregate (MTA). MTA has been shown to be the material of choice for regenerative endodontic procedures (Galler et al. 2016) and vital pulp therapy (European Society of Endodontology 2019) with a track record of improved clinical outcomes for direct pulp capping (Kundzina et al. 2017), partial pulpotomy (Qudeimat et al. 2007), revascularisation (Aly et al. 2019) and apical surgery (Kruse et al. 2016) and with similar outcomes to calcium hydroxide when used for indirect pulp capping (Leye Benoist et al. 2012; Koc Vural et al. 2017). The main shortcoming is the reported tooth discolouration (Parinyaprom et al. 2018; Aly et al. 2019) which affects the patient‐reported outcomes and quality of life and also necessitates further treatment. Tooth discolouration related to bismuth oxide use has been reported to occur in contact with collagen in dentine (Marciano et al. 2014), blood (Guimarães et al. 2015; Schembri Wismayer et al. 2016), formaldehyde (Marciano, Camilleri, et al. 2015, Berger et al. 2014) and sodium hypochlorite (Camilleri 2014; Camilleri et al. 2020) which is used as an antimicrobial irrigant in a number of clinical procedures. The discolouration moves from the material to the tooth structure by mineral migration across the interface (Marciano, Duarte, and Camilleri 2015) and is sustained even after an extended period of time (Krug et al. 2022, 2025) and care should be taken to avoid contact of materials that include bismuth oxide with the tooth structure. Leaching of bismuth from materials has been shown to occur to other neighbouring structures such as the skin of test animals (Schembri Wismayer et al. 2016). Percolation to the periapical tissues will negatively influence cell viability and gene expression associated with bismuth exposure. Contact with bismuth oxide results in metallothionein gene expression with upregulation of metallothionein (MT1 and MT2A) expression and down‐regulation of collagen‐1a (Col‐1a) and bone sialoprotein (BSP) expression (Pelepenko et al. 2024). The aim of this research is to investigate the interaction of bismuth oxide with sodium hypochlorite and characterise the phases formed using multiple methods.

## 2 Materials and Methods

Bismuth oxide (223 891, Sigma Aldrich, Gillingham, Kent, UK) was used in the current study as the control (C). It was characterised using various methods before and after exposure to 5.25% sodium hypochlorite (Cerkamed, Stalowa Wola, Poland) at different temperatures 20°C, 37°C and 60°C (B20, B37 and B60) for 24 weeks. Ten grams of bismuth oxide were mixed with 50 mL of sodium hypochlorite, and the solutions were continuously stirred over a hotplate. The solutions were changed weekly and after 24 weeks, they were discarded and the precipitate was washed with distilled water and dried in a desiccator using silica gel as a desiccant. The powders were stored in airtight containers awaiting further testing. Imaging by scanning electron microscopy to assess the grain and microstructure, optical microscopy for the colour changes, Fourier transform infrared (FT‐IR) spectroscopy for the compositional changes and X‐ray diffraction (XRD) for the phase changes was undertaken. Furthermore, more detailed phase analysis and X‐ray absorption of fine structure were done using synchrotron facilities. The X‐ray absorption near‐edge structure gives direct information about the valence state, and extended X‐ray absorption fine structure provides quantitative details about the local atomic structure thus enabling detailed information about the local electronic and atomic structure around a specific element in a material and a measurement of how X‐rays are absorbed near and above the core‐level binding energies of an element, indicating its formal oxidation state, the distances and types of neighbouring atoms, and their coordination numbers.

### 2.1 Scanning Electron Microscopy

C and B20, B37 and B60 were embedded in cold‐cure epoxy resin (Epoxy‐fix, Struers, Ballerup, Denmark) and the surfaces were polished with an automatic polishing machine (Buhler, Lake Buff, USA) using MD Piano 250, 500 and 1200 grit diamond discs under water coolant (Struers, Ballerup, Denmark) and polishing cloths—MD Largo, Dac, Nap (Struers) with ascending grades of diamond‐impregnated polishing liquids 9, 3 and 1 μm (Struers). The specimens were imaged under low pressure 60 Pa in a Phenom XL at 10–15 kV at 5 K and 15 K magnification.

### 2.2 Thermogravimetric Analysis

C and B20, B37 and B60 were assessed using thermogravimetric analysis/differential scanning calorimetry (TGA/DSC; Mettler‐Toledo, Beaumont Leys, Leicester, UK) at a temperature range of 40°C–800°C at a heating rate of 5°C min^−1^ and N_2_ flow rate of 40 mL min^−1^. Based on the TGA analysis, colour changes were observed at 450°C for B20, B37 and B60 and 2 additional groups after heating to 300°C and 500°C per temperature of test (20°C, 37°C, and 60°C) were created for further testing.

### 2.3 Optical Microscopy

All the specimens were imaged and photographed with a Nikon camera to record the color change of the hypochlorite‐treated materials at different temperatures compared to the untreated bismuth oxide (C).

### 2.4 Fourier‐Transform Infrared Spectroscopy

Two to five milligrams of the samples were mixed with 100 mg potassium bromide (Acros Organics, 99 + %, IR grade). Pellets were formed between dry stainless‐steel plates at 1 MPa and retrieved after at least 30 s standing. FT‐IR was performed (Spectrum II, Perkin Elmer) in transmittance mode over the range 400–4000 cm^−1^, with a resolution of 2.0 cm^−1^ and using 80 scans per sample.

### 2.5 Phase Analysis

Phase analysis of all specimens was performed by X‐ray diffraction using a D8 Advance (Bruker) with Cu Kα radiation at 40 mA and 45 kV over 10°–60° 2θ, stepping 0.02° per 0.6 s. A Pilatus area detector was used to measure X‐ray diffraction from the samples at 13 keV X‐ray energy. XRD data were calibrated using the XRD spectrum of a Si pellet measured at 13 keV X‐ray energy.

### 2.6 X‐Ray Absorption Fine Structure (XAFS)

Calculated amounts of each sample were mixed with boron nitride using a mortar and pestle to form a homogeneous mixture and pressed into pellets. The pellets were loaded onto 3D‐printed sample racks and mounted on beamline B18 at Diamond Light Source, UK (Dent et al. 2009). A Si (111) double crystal monochromator was used for energy selection. The incident and transmitted beam intensities were measured using ionisation chambers. Energy calibration was performed using a Bi metal foil. Bi L3 edge (13.419 keV) XAFS spectra were measured in transmission mode on all the samples, and also on relevant Bi reference standards (Bi metal, Bi(OH)_3_, NaBiO_3_) for comparison.

## 3 Results

### 3.1 Scanning Electron Microscopy

**FIGURE 1:**
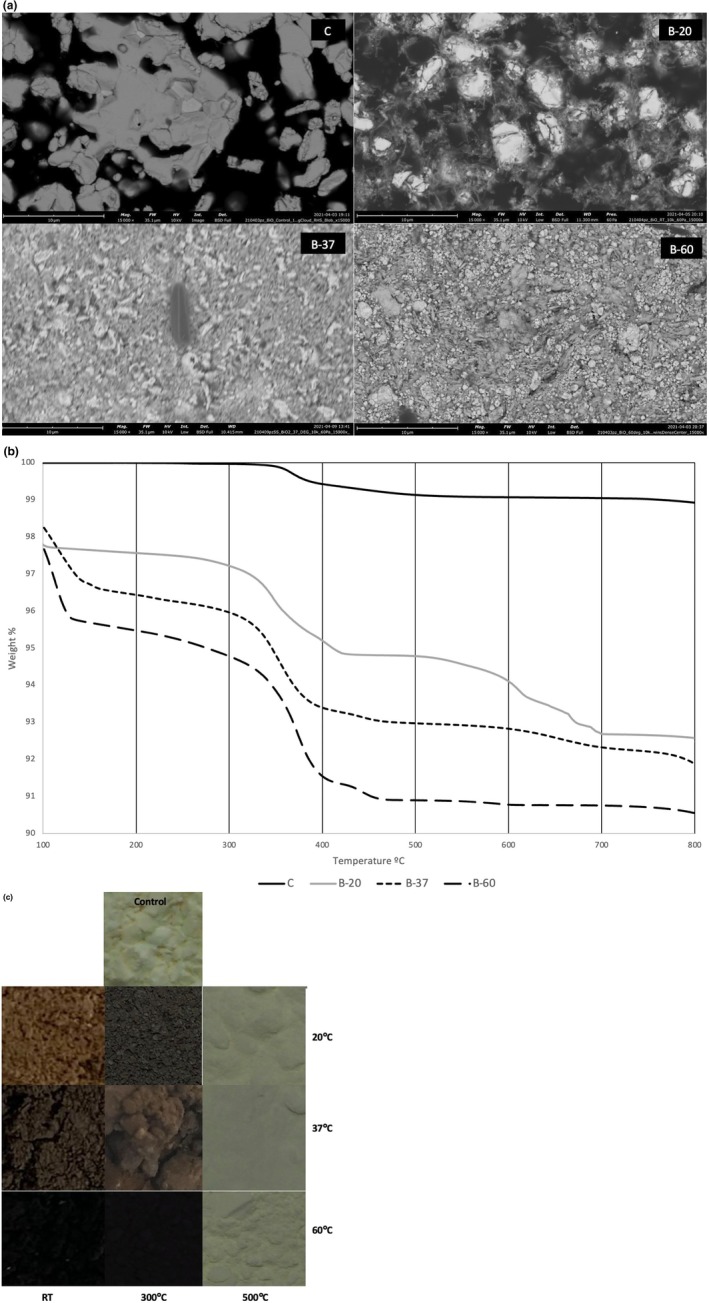
(a) Scanning electron micrographs of the materials showing the changes in microstructure with exposure to sodium hypochlorite at different temperatures over a period of 24 weeks. Mag: 15 K x. (b) Thermographic analysis showing the weight changes of the untreated bismuth oxide and powders of bismuth oxide in sodium hypochlorite prepared at different temperatures. A significant weight loss is shown between 300°C and 500°C. (c) Bismuth oxide imaged after immersion in sodium hypochlorite for 24 weeks at different temperatures followed by placement in oven for 30 min at either 300°C or 500°C showing the changes in colour with darkening in contact with sodium hypochlorite and yellow colour resumed after heating to 500°C.

The scanning electron micrographs (Figure 1) show a change in grain microstructure at the different temperatures with smaller particle sizes produced at higher temperatures. The finer grain boundaries for the materials prepared at higher temperatures were evident.

### 3.2 Thermographic Analysis

The results of the thermographic analysis (Figure 1b) reveal a distinct change in mass at ~450°C with the control (C) showing the least drop in weight compared to the other materials tested (B20, B37, B60). The change in mass was also accompanied by a visible colour change which was investigated further. All the specimens were either heated to 300°C or 500°C. creating subgroups for each temperature tested.

### 3.3 Optical Microscopy

The colour changes shown in Figure 1c ranged from yellow, orange to brown and darker brown with increasing temperatures. All the specimens reverted back to yellow from dark brown/black when heated to 500°C.

### 3.4 Fourier‐Transform Infrared Spectroscopy

**FIGURE 2:**
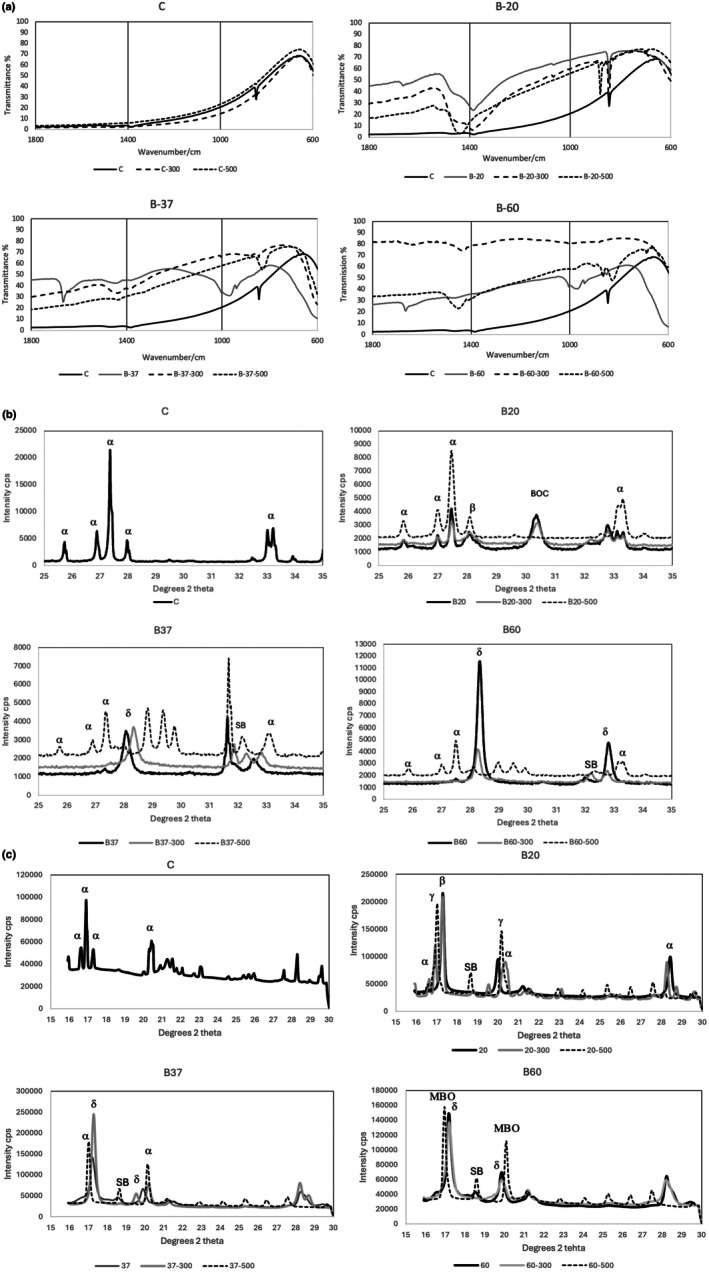
(a) Fourier transform infrared spectrograms of the control bismuth oxide and bismuth oxide mixed with sodium hypochlorite at different temperatures (20°C, 37°C, 60°C) for 24 weeks showing the phase changes induced by the sodium hypochlorite after 24 weeks of exposure and also after heating to either 300°C or 500°C compared to the unmodified bismuth oxide. The spectrograms show the changes between 600 and 1000 cm^−1^. (b) XRD plots obtained by Bragg–Brentano configuration and (c): Plots from the Pilatus detector of the control bismuth oxide and bismuth oxide mixed with sodium hypochlorite at different temperatures (20°C, 37°C, 60°C) for 24 weeks showing the phase changes induced by the sodium hypochlorite and those by the heating phases. The plots show intensity in counts per second plotted against diffractometer angle °2θ from 20° to 40°2θ. The phases present are the following—monoclinic α‐Bi_2_O_3_ (ICDD: 01‐070‐8243); δ‐Bi_2_O_3_ (ICDD: 00‐052‐1007); β‐Bi_2_O_3_ (ICDD: 01‐077‐5341); (BOC) orthorhombic bismuth oxycarbonate (ICDD: 01‐070‐8631); (SB) rhombohedral hexagonal sodium bismuthate (ICDD: 01‐070‐6347).

The FT‐IR spectrograms are shown in Figure 2a. The changes observed between 600 and 1000 cm^−1^ are shown with the bismuth oxide pattern modified when mixed with sodium hypochlorite and the pattern changes intensified with an increase in temperature. The control group showed minimal changes when heated to 300°C and 500°C. At room temperature, it exhibited a peak at 840 cm^−1^, indicating Bi‐O stretching which disappeared after heating to higher temperatures eliminating the impurities. This was also evident in B20 and B20‐300. None of the samples recovered completely the bismuth oxide structure (C) on being heated to 500°C. Note that the peak at 825 cm^−1^ in B37‐500 and B60‐500 is an unknown phase.

### 3.5 Phase Analysis

The XRD plots for the Bragg–Brentano configuration are shown in Figure 2b and the data collected on B18, Diamond Light Source, using a Pilatus detector in Figure 2c. For both configurations the control bismuth oxide exhibited peaks for monoclinic α‐bismuth oxide (ICDD: 01‐070‐8243). The interaction of bismuth oxide with sodium hypochlorite produced different polymorphs depending on temperature. The β‐Bi_2_O_3_ (ICDD: 01‐077‐5341) was predominant at 20°C with remnants of the α‐phase indicating incomplete reaction. Bismuth oxycarbonate (ICDD: 01‐070‐8631) and sodium bismuthate (ICDD: 01‐070‐6347) were also present. At 37°C and 60°C, the material was a mixture of δ‐phase (ICDD: 00‐052‐1007) and sodium bismuthate. Heating to 300°C maintained the same phases but at 500°C there was conversion to α‐phase for the B37, a metastable bismuth oxide for B60 and γ‐phase for the B20. Some unidentified phases are shown in the B37 and B60 which are shown as unlabeled in Figure 2b.

### 3.6 X‐Ray Absorption Fine Structure (XAFS)

**FIGURE 3:**
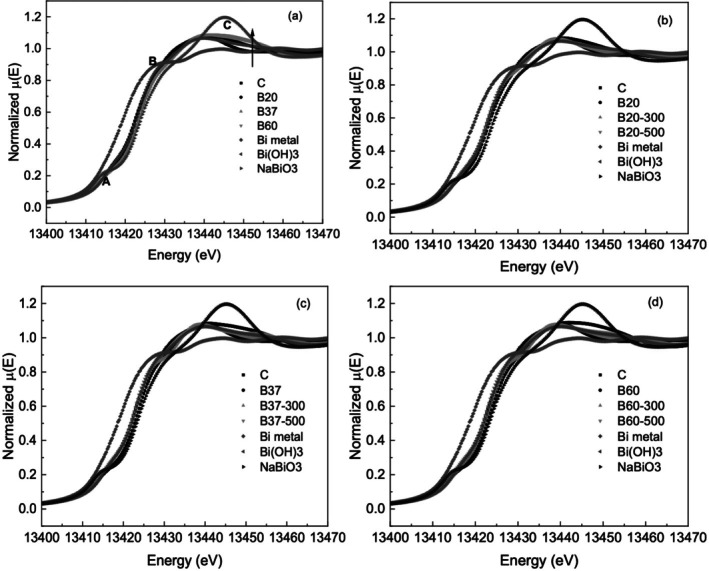
XANES spectra for (a) C, B20, B37, B60 samples, (b–d) specimens heated to 300°C and 500°C. Bi_2_O_3_‐calcined (α‐Bi_2_O_3_) Bi metal foil, Bi(OH)_3_, and NaBiO_3_ are plotted for reference.

The XANES spectra for all the samples are plotted in Figure 3, in comparison with Bi metal, Bi(OH)_3_ and NaBiO_3_. No significant changes were observed in the edge energy position implying that the oxidation state of Bi remains unchanged between the samples (Figure 3a). Slight differences were observed in the XANES with increases in temperature from B20 to B60, with the rising of a shoulder which is attributed to changes in the chemical environment and symmetry around Bi. Significant changes were observed in the XANES spectra for B20, B37 and B60 when heated to 300°C and 500°C (Figure 3b–d), indicating changes to the local structure around Bi.

**FIGURE 4:**
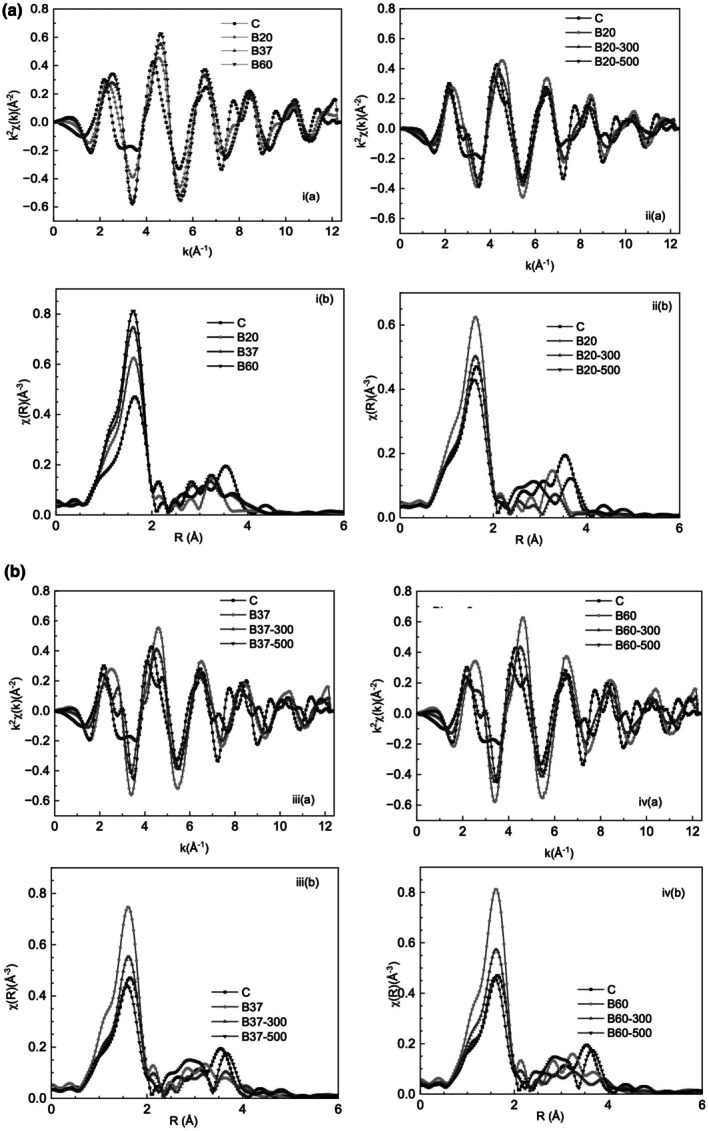
k^2^‐weighted EXAFS data (a) and their Fourier Transforms (b) over the range k = 3–11.5 Å^−1^ for (i) C, B20, B37, B60 samples and (ii)–(v) specimens heated to 300°C and 500°C.

The EXAFS spectra measured on all the samples are presented in Figure 4, in comparison with Bi metal, Bi(OH)_3_ and NaBiO_3_. The first peak in the Fourier Transform (1–2 Å) is due to Bi‐O scattering pairs. Changes observed in the first peak in the Fourier‐transformed EXAFS spectra for all samples reflect the changes to the average Bi‐O first coordination shell, which are the result of the presence of mixed phases of Bi_2_O_3_ in the samples. The various theoretical Bi‐O bond lengths are summarised in Table 1. EXAFS fitting results are summarised in the Table 2.

**TABLE 1:** Crystal structures of the different phases of bismuth oxide, and the nearest bismuth‐oxygen bond distances. NaBiO_3_ is also included. Wherever there are inequivalent Bi sites, they are listed as Bi1, Bi2, etc.

	Crystal structure	Space group	Bi‐O bond distances
α‐Bi_2_O_3_	Monoclinic	P21/c	Bi1: 2.075 Å, 2.174 Å, 2.209 Å, 2.548 Å, 2.636 Å Bi2: 2.136 Å, 2.224 Å, 2.290 Å, 2.477 Å, 2.531 Å Average bond distances: 3 × 2.139 Å, 2 × 2.548 Å
β‐Bi_2_O_3_	Tetragonal	P‐42_1c	2.096 Å, 2.128 Å, 2.253 Å, 2.463 Å Average bond distances: 3 × 2.159 Å, 1 × 2.463 Å
γ‐Bi_2_O_3_	Cubic (bcc)	I23	Bi1: 2.087 Å, 2.234 Å, 2.283 Å, 2.595 Å, 2.622 Å Avg = 2.201, 2.608 [3 + 2] Bi2: 1.910 Å, 1.910 Å, 1.910 Å, 1.910 Å. Average = 1.910 Å [4] Average bond distances: 1.910*4, 2.201*3, 2.608*2
δ‐Bi_2_O_3_	Cubic (fcc)	Fm‐3 m	2.451 *4
NaBiO_3_	Trigonal	‐R3	2.094*3, 2.137*3 Average bond distances = 2.115*6

**TABLE 2:** EXAFS fit results for specimens tested. A structural model was constructed for the Bi‐O coordination shell using FEFF6.1 (Newville 2001). The fitting was performed over k = 3–11.5 Å^−1^, k^w^(w = 1,2,3), *R* = 1–2.2 Å. The model parameters were allowed to vary while fitting to yield the best‐fit values for bond lengths (R), coordination numbers (N) and Debye–Waller factors (σ^2^).

	N	σ^2^ (Å^2^)	R(Å)
B20	3.1 (2)	0.0049 (7)	2.120 (3)
B37	3.6 (2)	0.0050 (5)	2.107 (2)
B60	3.8 (2)	0.0047 (6)	2.103 (2)
B20	3.1 (2)	0.0050 (9)	2.119 (3)
B20‐300	2.6 (2)	0.005 (1)	2.125 (4)
B20‐500	2.5 (4)	0.007 (2)	2.127 (6)
B37	3.6 (2)	0.0050 (5)	2.107 (2)
B37‐300	2.7 (2)	0.0049 (8)	2.113 (3)
B37‐500	2.7 (3)	0.007 (1)	2.103 (4)
B60	3.8 (2)	0.0047 (6)	2.103 (2)
B60‐300	3.1 (3)	0.0059 (7)	2.122 (2)
B60‐500	2.6 (3)	0.006 (1)	2.098 (4)

## 4 Discussion

The current research investigated the microstructural, physical and chemical changes of bismuth oxide in contact with sodium hypochlorite. The interaction of bismuth oxide with sodium hypochlorite occurs clinically when sodium hypochlorite is used to reduce the microbial load, and the bismuth oxide is included in dental materials as a radiopacifier. The changes observed are shown in the graphical abstract. The tooth discolouration caused by mineral trioxide aggregate was initially reported in clinical observations (Felman and Parashos 2013; Lenherr et al. 2012) and in laboratory investigations (Vallés et al. 2013, 2015; Camilleri 2014; Kang et al. 2015). Light and anaerobic conditions were observed to cause the change in colour (Vallés et al. 2013, 2015; Kang et al. 2015). Sodium hypochlorite was implicated (Camilleri 2014) and the change was initially attributed to the formation of sodium bismuthate caused by the destabilisation of the bismuth oxide reacting with the sodium hypochlorite (Camilleri et al. 2020). To enable a better understanding of the interactions, further research was undertaken to assess the phase changes which were dependent on the temperature. The knowledge of these changes is imperative as the mitigation of the tooth discolouration could be through the ability to reverse the reaction. Alternative radiopacifiers have been tested to replace the bismuth oxide but none impart the necessary radiopacity. Similar radiopacity can only be achieved by increasing the radiopacifier loading (Camilleri 2010; Tanomaru‐Filho et al. 2012) and this will necessitate the reduction in cement amount (Cahyanto et al. 2023; Cardinali and Camilleri 2023) to levels where there will not be enough cement (binder) to hold the material together resulting in an inability to set as has been reported (Raman and Camilleri 2024).

The effect of temperature on the phase changes was also investigated with the selection of a range of temperatures namely room temperature and body temperature, to be able to compare the data with the literature available on bismuth oxide used in industry, as well as at 60°C which was selected as a way to accelerate the reaction This is important because bismuth oxide in dental materials may be subject to a rise in temperature during warm vertical compaction (Atmeh et al. 2020) or when heating irrigating solutions (Stojicic et al. 2010; Wright et al. 2020).

The initial work conducted (Camilleri 2014) had an immersion time of 24 h which showed the colour change but not the phase changes. In a later study, sodium bismuthate was identified when the bismuth oxide was tested after 1, 4, 12 and 24 weeks with the experiment being undertaken at room temperature. The current work created a range of temperatures for the postulation that the phase changes are temperature dependent. The 24 weeks were retained to enable comparisons to previous work (Camilleri et al. 2020).

To ensure that all the changes were identified, a range of tests were undertaken. The combination of the methods allowed a complete analysis of the structural and chemical changes of the bismuth oxide in contact with sodium hypochlorite and the changes were accelerated with a rise in temperature. The scanning electron microscopy enabled the imaging of the material microstructure at a very high power to show the grain boundaries and also the particle size which changed with the change in phase. The imaging using the light microscope captured the colour changes. Compositional changes were possible with FT‐IR spectroscopy as a comparison of the change in chemistry could be made when adding the sodium hypochlorite. Phase changes were recorded using X‐ray diffraction with a Bragg–Brentano geometry and also using a Pilatus detector on the beam line. The Bragg Brentano geometry enables the detection of the different phases of the bismuth oxide. The Pilatus detector in the beamline is a type of photon‐counting hybrid pixel detector featuring a single‐photon counting with a low‐energy threshold thus enabling background suppression for small‐angle scattering at synchrotrons. The data were collected at the 13 KeV X‐ray energy. Bismuth oxide's ability to shield or attenuate radiation can also be measured at 16 KeV photon energy. XAFS was used to determine the average local structure around Bi and its changes resulting from interaction with sodium hypochlorite. Analysis of the XAFS traditionally breaks up into two parts: XANES (X‐ray absorption near‐edge structure) and EXAFS (extended X‐ray absorption fine structure) (Dent et al. 2009). XANES refers to the region close to the edge (within ~30 eV) and is sensitive to the chemical state of the excited atom. EXAFS refers to the oscillations well above the edge (30 eV) and gives local structural information about the excited atom. XAFS data analysis was performed using the Demeter package (Koningsberger and Prins 1988). The data were processed and the extracted EXAFS oscillations, χ (k), were Fourier‐transformed into real space χ (R) for fitting. $k=\sqrt{\frac{2m\left(E-E_{0}\right)}{\hbar^{2}}}$ where m is the electron mass and E_0_ is the edge energy of the relevant absorption edge.

The XANES spectra of Bi compounds at the L3 edge are characterised by three main features: A, B and C (Figure 3). Feature A is due to the 2p_3/2_ ➔6 s transition; features B and C are due to the 2p_3/2_ ➔ 6d transition (Jiang and Spence 2006). The 6 s level is empty in Bi^5+^, and therefore feature A is characteristic of Bi^5+^, as is the case for NaBiO_3_. In contrast, the 6 s level is filled in both Bi^(0)^ and Bi^3+^, so this feature is either completely absent (Bi^(0)^), or very weak (Bi^3+^). The absence of a prominent feature A suggests that Bi has an average oxidation state lower than 5+ in all of the specimens tested. However, this does not rule out the presence of Bi^5+^, as due to the limited availability of reference standards (arising from the complicated chemistry of the interaction of bismuth oxide with NaOCl), it was not possible to reliably perform a linear combination analysis of the XANES spectra of all the samples.

The XRD analysis showed the samples were a mixture of different polymorphs of Bi_2_O_3_: α‐Bi_2_O_3_ (monoclinic structure), β‐Bi_2_O_3_ (tetragonal structure), γ‐Bi_2_O_3_ (body centered cubic structure), and δ‐Bi_2_O_3_ (face centered cubic structure) (Malmros 1970; Blower and Greaves 1988; Harwig 1978; Kumada et al. 2000). Only the phase is stable at room temperature. The δ phase bismuth oxide is stable at higher temperatures (730°C–825°C) (Deng et al. 2011). The reaction with sodium hypochlorite enabled the phase changes to occur at room temperature and different phases formed at different temperatures. Besides the polymorphs, other phases were also formed. The formation of the bismuth oxycarbonate by the combination of bismuth oxide and carbon dioxide in the presence of moisture has been previously reported at room temperature (Ortiz Quiñonez et al. 2018). The oxycarbonate gives a light brown hue to the bismuth oxide which is yellow in colour as shown in the optical microscopy images.

The current study shows the phase changes with the formation of multiple polymorphs of bismuth oxide when in contact with sodium hypochlorite. It also shows that the black colour is caused by the δ‐phase, and the reaction occurs at room temperature which has so far never been reported. Also unreported is the conversion to the α‐phase on heating to 500°C. The bismuth oxide polymorphism has been previously reported but it occurred at higher temperatures. The current study also investigated phase and compositional changes at 300°C and 500°C, as the bismuth and hypochlorite mixture exhibited loss of mass and a colour change when TGA was undertaken. The phase changes at 450°C have never been reported. The heating also restored the yellow colour of the α‐phase.

Other reactions occurred with the formation of sodium bismuthate. These reactions have already been reported in the dentistry context (Camilleri et al. 2020). Bismuth oxide in the presence of a strong alkali such as sodium hydroxide can form bismuth hydroxide and in the presence of bromine sodium bismuthate is formed (von Brauer 1975). A similar reaction occurred in the current experiment with the oxidation of Bi(III) to Bi(V) in the presence of sodium hypochlorite which dissociated liberating free chlorine. In the current study, sodium bismuthate was shown to be present in the mixtures of bismuth oxide and sodium hypochlorite prepared at 37°C and 60°C and heated at 300°C. Sodium bismuthate was detected in earlier analysis of similar mixtures (Camilleri et al. 2020). The δ‐phase was present in both the B37 and B60. This phase gives the black discolouration seen in the optical images (Fujita et al. 2020; Nguyen and Edalati 2024). This phase is a defective face‐centered cubic which is rich in oxygen vacancies and can thus be used effectively as an oxygen conductor. The presence of this phase at room and body temperature and its contribution to tooth discolouration has so far never been reported.

## 5 Conclusions and Recommendations

The research undertaken explains the colour changes of bismuth oxide in contact with sodium hypochlorite. The δ‐phase bismuth oxide formation led to the material darkening which will cause tooth discolouration in a clinical setting. Due to the phase changes, the material chemistry after the interaction is different from that of the material placed in the tooth. The by‐products of the reaction have not been tested for use in patients. It is recommended to ban the use of bismuth oxide from dental materials and other clinical use due to its instability. The clinical guidance for endodontic treatment needs to be changed to reflect this. The reaction can be reversed at high temperatures which is not relevant in the clinical context.

## Author Contributions

Josette Camilleri: contributed to conception and design, contributed to analysis, drafted the manuscript, critically reviewed the manuscript, gave final approval, agrees to be accountable for all aspects of work ensuring integrity and accuracy. Paul Zaslansky: contributed to analysis, critically reviewed the manuscript, gave final approval, agrees to be accountable for all aspects of work ensuring integrity and accuracy. Nitya Ramanan: contributed to analysis, drafted the manuscript, critically reviewed the manuscript, gave final approval, agrees to be accountable for all aspects of work ensuring integrity and accuracy.

## Funding

The authors have nothing to report.

## Conflicts of Interest

The authors declare no conflicts of interest.

## Supporting information


**Data S1:** Supporting Information.

## Data Availability

The data that support the findings of this study are openly available in University of Birmingham at https://doi.org/10.25500/edata.bham.00001309

## References

[iej70077-bib-0001] Aly, M. M. , S. E. E. Taha , M. A. el Sayed , R. Youssef , and H. M. Omar . 2019. “Clinical and Radiographic Evaluation of Biodentine and Mineral Trioxide Aggregate in Revascularization of Non‐Vital Immature Permanent Anterior Teeth (Randomized Clinical Study).” International Journal of Paediatric Dentistry 29: 464–473. 30702789 10.1111/ipd.12474

[iej70077-bib-0002] Atmeh, A. R. , M. Hadis , and J. Camilleri . 2020. “Real‐Time Chemical Analysis of Root Filling Materials With Heating: Guidelines for Safe Temperature Levels.” International Endodontic Journal 53: 698–708. 31955442 10.1111/iej.13269

[iej70077-bib-0003] Berger, T. , A. Z. Baratz , and J. L. Gutmann . 2014. “In Vitro Investigations Into the Etiology of Mineral Trioxide Tooth Staining.” Journal of Conservative Dentistry 17: 526–530. 25506138 10.4103/0972-0707.144584 PMC4252924

[iej70077-bib-0004] Blower, S. K. , and C. Greaves . 1988. “The Structure of β‐Bi_2_O_3_ From Powder Neutron Diffraction Data.” Acta Crystallographica C44: 587.

[iej70077-bib-0005] Cahyanto, A. , P. Rath , T. X. Teo , et al. 2023. “Designing Calcium Silicate Cements With on‐Demand Properties for Precision Endodontics.” Journal of Dental Research 102: 1425–1433. 37861249 10.1177/00220345231198185

[iej70077-bib-0008] Camilleri, J. 2010. “Evaluation of the Physical Properties of an Endodontic Portland Cement Incorporating Alternative Radiopacifiers Used as Root‐End Filling Material.” International Endodontic Journal 43: 231–240. 20158535 10.1111/j.1365-2591.2009.01670.x

[iej70077-bib-0007] Camilleri, J. 2014. “Color Stability of White Mineral Trioxide Aggregate in Contact With Hypochlorite Solution.” Journal of Endodontics 40: 436–440. 24565667 10.1016/j.joen.2013.09.040

[iej70077-bib-0006] Camilleri, J. , J. Borg , D. Damidot , et al. 2020. “Colour and Chemical Stability of Bismuth Oxide in Dental Materials With Solutions Used in Routine Clinical Practice.” PLoS One 15: e0240634. 33176336 10.1371/journal.pone.0240634 PMC7657490

[iej70077-bib-0009] Cardinali, F. , and J. Camilleri . 2023. “A Critical Review of the Material Properties Guiding the Clinician's Choice of Root Canal Sealers.” Clinical Oral Investigations 27: 4147–4155. 37460901 10.1007/s00784-023-05140-w PMC10415471

[iej70077-bib-0010] Council on Dental Materials, Instruments, and Equipment . 1981. “The Desirability of Using Radiopaque Plastics in Dentistry: A Status Report.” Journal of the American Dental Association 102: 347–349. 6936478 10.14219/jada.archive.1981.0058

[iej70077-bib-0011] Deng, H. Y. , W. C. Hao , and H. Z. Xu . 2011. “A Transition Phase in the Transformation From α‐, 𝛽‐ and 𝜀‐ to 𝛿‐Bismuth Oxide.” Chinese Physics Letters 28: 056101.

[iej70077-bib-0012] Dent, A. J. , G. Cibin , S. Ramos , et al. 2009. “B18: A Core XAS Spectroscopy Beamline for Diamond.” Journal of Physics: Conference Series 190: 012039.

[iej70077-bib-0013] European Commission . 2023. “Proposal for a Regulation of the European Parliament and of the Council Amending Regulations (EU) 2017/745 and (EU) 2017/746 as Regards the Transitional Provisions for Certain Medical Devices and in vitro diagnostic medical devices, 1–14”.

[iej70077-bib-0014] European Society of Endodontology , Duncan H. F. , and K. M. Galler 2019. “European Society of Endodontology Position Statement: Management of Deep Caries and the Exposed Pulp.” International Endodontic Journal 52: 923–934. 30664240 10.1111/iej.13080

[iej70077-bib-0015] Felman, D. , and P. Parashos . 2013. “Coronal Tooth Discoloration and White Mineral Trioxide Aggregate.” Journal of Endodontics 39: 484–487. 23522541 10.1016/j.joen.2012.11.053

[iej70077-bib-0016] Fujita, I. , P. Edalati , Q. Wang , et al. 2020. “Novel Black Bismuth Oxide (Bi_2_O_3_) With Enhanced Photocurrent Generation, Produced by High‐Pressure Torsion Straining.” Scripta Materialia 187: 366–370.

[iej70077-bib-0017] Galler, K. M. , G. Krastl , S. Simon , et al. 2016. “European Society of Endodontology Position Statement: Revitalization Procedures.” International Endodontic Journal 49: 717–723. 26990236 10.1111/iej.12629

[iej70077-bib-0018] Guimarães, B. M. , T. Tartari , M. A. Marciano , et al. 2015. “Color Stability, Radiopacity, and Chemical Characteristics of White Mineral Trioxide Aggregate Associated With 2 Different Vehicles in Contact With Blood.” Journal of Endodontics 41: 947–952. 25799532 10.1016/j.joen.2015.02.008

[iej70077-bib-0019] Harwig, H. A. 1978. “On the Structure of Bismuthsesquioxide: The α, β, γ, and δ‐Phase.” Zeitschrift für Anorganische und Allgemeine Chemie 444: 151–166.

[iej70077-bib-0020] Jiang, N. , and J. C. H. Spence . 2006. “Can Near‐Edge Structure of the bi L3 Edge Determine the Formal Valence States of Bi?” Journal of Physics. Condensed Matter 18: 8029–8036.

[iej70077-bib-0021] Kang, S. H. , Y. S. Shin , H. S. Lee , et al. 2015. “Color Changes of Teeth After Treatment With Various Mineral Trioxide Aggregate‐Based Materials: An Ex Vivo Study.” Journal of Endodontics 41: 737–741. 25732402 10.1016/j.joen.2015.01.019

[iej70077-bib-0022] Koc Vural, U. , A. Kiremitci , and S. Gokalp . 2017. “Randomized Clinical Trial to Evaluate MTA Indirect Pulp Capping in Deep Caries Lesions After 24‐Months.” Operative Dentistry 42: 470–477. 28581920 10.2341/16-110-C

[iej70077-bib-0023] Koningsberger, D. C. , and R. Prins . 1988. X‐Ray Absorption: Principles, Applications and Techniques of EXAFS, SEXAFS and XANES. Wiley.

[iej70077-bib-0024] Krug, R. , C. Ortmann , S. Reich , B. Hahn , G. Krastl , and S. Soliman . 2022. “Tooth Discoloration Induced by Apical Plugs With Hydraulic Calcium Silicate‐Based Cements in Teeth With Open Apices—A 2‐Year In Vitro Study.” Clinical Oral Investigations 26: 375–383. 34151389 10.1007/s00784-021-04009-0 PMC8791895

[iej70077-bib-0025] Krug, R. , F. Schwarz , B. Hahn , et al. 2025. “Long‐Term Tooth Discoloration Induced by Apical Plugs With Hydraulic Calcium Silicate‐Based Cements in Bovine Teeth With Open Apices—A 6‐Year In‐Vitro Study.” Clinical Oral Investigations 29: 332. 40467906 10.1007/s00784-025-06407-0 PMC12137506

[iej70077-bib-0026] Kruse, C. , R. Spin‐Neto , R. Christiansen , A. Wenzel , and L. L. Kirkevang . 2016. “Periapical Bone Healing After Apicectomy With and Without Retrograde Root Filling With Mineral Trioxide Aggregate: A 6‐Year Follow‐Up of a Randomized Controlled Trial.” Journal of Endodontics 42: 533–537. 26898567 10.1016/j.joen.2016.01.011

[iej70077-bib-0027] Kumada, N. , N. Kinomura , and A. W. Sleight . 2000. “Neutron Powder Diffraction Refinement of Ilmenite‐Type Bismuth Oxides: ABiO_3_ (A = Na, ag).” Materials Research Bulletin 35: 2397–2402.

[iej70077-bib-0028] Kundzina, R. , L. Stangvaltaite , H. M. Eriksen , and E. Kerosuo . 2017. “Capping Carious Exposures in Adults: A Randomized Controlled Trial Investigating Mineral Trioxide Aggregate Versus Calcium Hydroxide.” International Endodontic Journal 50: 924–932. 27891629 10.1111/iej.12719

[iej70077-bib-0029] Lenherr, P. , N. Allgayer , R. Weiger , A. Filippi , T. Attin , and G. Krastl . 2012. “Tooth Discoloration Induced by Endodontic Materials: A Laboratory Study.” International Endodontic Journal 45: 942–949. 22506849 10.1111/j.1365-2591.2012.02053.x

[iej70077-bib-0030] Leye Benoist, F. , F. Gaye Ndiaye , A. W. Kane , H. M. Benoist , and P. Farge . 2012. “Evaluation of Mineral Trioxide Aggregate (MTA) Versus Calcium Hydroxide Cement (Dycal()) In the Formation of a Dentine Bridge: A Randomised Controlled Trial.” International Dental Journal 62: 33–39. 22251035 10.1111/j.1875-595X.2011.00084.x PMC9374926

[iej70077-bib-0031] Malmros, G. 1970. “The Crystal Structure of Alpha‐Bi_2_O_2_ .” Acta Chemica Scandinavica 24: 384–396.

[iej70077-bib-0032] Marciano, M. A. , J. Camilleri , R. F. Lia Mondelli , et al. 2015. “Potential Dental Staining of Root Canal Sealers With Formulations Containing Bismuth Oxide and Formaldehyde.” ENDO‐Endodontic Practice Today 9: 39–45.

[iej70077-bib-0034] Marciano, M. A. , R. M. Costa , J. Camilleri , R. F. Mondelli , B. M. Guimarães , and M. A. Duarte . 2014. “Assessment of Color Stability of White Mineral Trioxide Aggregate Angelus and Bismuth Oxide in Contact With Tooth Structure.” Journal of Endodontics 40: 1235–1240. 25069940 10.1016/j.joen.2014.01.044

[iej70077-bib-0035] Marciano, M. A. , M. A. Duarte , and J. Camilleri . 2015. “Dental Discoloration Caused by Bismuth Oxide in MTA in the Presence of Sodium Hypochlorite.” Clinical Oral Investigations 19: 2201–2209. 25922130 10.1007/s00784-015-1466-8

[iej70077-bib-0049] Newville, M. 2001. “IFEFFIT: Interactive XAFS Analysis and FEFF Fitting.” Journal of Synchrotron Radiation 8: 322–324. 11512767 10.1107/s0909049500016964

[iej70077-bib-0036] Nguyen, T. T. , and K. Edalati . 2024. “Efficient Photocatalytic Hydrogen Production on Defective and Strained Black Bismuth (III) Oxide.” International Journal of Hydrogen Energy 96: 841–848.

[iej70077-bib-0037] Ortiz Quiñonez, J. L. , C. Vega Verduga , D. Díaz , and I. Zumeta Dubé . 2018. “Transformation of Bismuth and β‐Bi_2_O_3_ Nanoparticles Into (BiO)_2_CO_3_ and (BiO)_4_(OH)_2_CO_3_ by Capturing CO_2_: The Role of Halloysite Nanotubes and “Sunlight” on the Crystal Shape and Size.” Crystal Growth & Design 18: 4334–4346.

[iej70077-bib-0038] Parinyaprom, N. , A. Nirunsittirat , P. Chuveera , et al. 2018. “Outcomes of Direct Pulp Capping by Using Either Proroot Mineral Trioxide Aggregate or Biodentine in Permanent Teeth With Carious Pulp Exposure in 6‐ To 18‐Year‐Old Patients: A Randomized Controlled Trial.” Journal of Endodontics 44: 341–348. 29275850 10.1016/j.joen.2017.10.012

[iej70077-bib-0039] Pelepenko, L. E. , M. A. Marciano , R. M. Shelton , and J. Camilleri . 2024. “Leaching and Cytotoxicity of Bismuth Oxide in ProRoot MTA—A Laboratory Investigation.” International Endodontic Journal 57: 1293–1314. 38804676 10.1111/iej.14101

[iej70077-bib-0040] Qudeimat, M. A. , K. M. Barrieshi‐Nusair , and A. I. Owais . 2007. “Calcium Hydroxide vs Mineral Trioxide Aggregates for Partial Pulpotomy of Permanent Molars With Deep Caries.” European Archives of Paediatric Dentistry 8: 99–104. 17555692 10.1007/BF03262577

[iej70077-bib-0041] Raman, V. , and J. Camilleri . 2024. “Characterization and Assessment of Physical Properties of 3 Single Syringe Hydraulic Cement‐Based Sealers.” Journal of Endodontics 50: 381–388. 38219956 10.1016/j.joen.2024.01.001

[iej70077-bib-0042] Schembri Wismayer, P. , C. Y. Lung , F. Rappa , F. Cappello , and J. Camilleri . 2016. “Assessment of the Interaction of Portland Cement‐Based Materials With Blood and Tissue Fluids Using an Animal Model.” Scientific Reports 29, no. 6: 34547. 10.1038/srep34547 PMC5041115 27683067

[iej70077-bib-0043] Stojicic, S. , S. Zivkovic , W. Qian , H. Zhang , and M. Haapasalo . 2010. “Tissue Dissolution by Sodium Hypochlorite: Effect of Concentration, Temperature, Agitation, and Surfactant.” Journal of Endodontics 36: 1558–1562. 20728727 10.1016/j.joen.2010.06.021

[iej70077-bib-0044] Tanomaru‐Filho, M. , V. Morales , G. F. da Silva , et al. 2012. “Compressive Strength and Setting Time of MTA and Portland Cement Associated With Different Radiopacifying Agents.” ISRN Dentistry 2012: 898051. 22957262 10.5402/2012/898051 PMC3432372

[iej70077-bib-0045] Vallés, M. , M. Mercadé , F. Duran‐Sindreu , J. L. Bourdelande , and M. Roig . 2013. “Influence of Light and Oxygen on the Color Stability of Five Calcium Silicate‐Based Materials.” Journal of Endodontics 39: 525–528. 23522550 10.1016/j.joen.2012.12.021

[iej70077-bib-0046] Vallés, M. , M. Roig , F. Duran‐Sindreu , S. Martínez , and M. Mercadé . 2015. “Color Stability of Teeth Restored With Biodentine: A 6‐Month In Vitro Study.” Journal of Endodontics 41: 1157–1160. 25937179 10.1016/j.joen.2015.03.014

[iej70077-bib-0047] von Brauer, G. 1975. Handbuch der Präparativen Anorganischen Chemie/1, edited by M. Baudler , 604. Enke.

[iej70077-bib-0048] Wright, P. P. , B. Kahler , and L. J. Walsh . 2020. “The Effect of Temperature on the Stability of Sodium Hypochlorite in a Continuous Chelation Mixture Containing the Chelator Clodronate.” Australian Endodontic Journal 46: 244–248. 32129922 10.1111/aej.12399

